# Keeping the Spirits Up: The Effect of Teachers’ and Parents’ Emotional Support on Children’s Working Memory Performance

**DOI:** 10.3389/fpsyg.2017.00512

**Published:** 2017-04-04

**Authors:** Loren Vandenbroucke, Jantine Spilt, Karine Verschueren, Dieter Baeyens

**Affiliations:** ^1^Parenting and Special Education Unit, Faculty of Psychology and Educational Sciences, KU LeuvenLeuven, Belgium; ^2^School Psychology and Child and Adolescent Development, Faculty of Psychology and Educational Sciences, KU LeuvenLeuven, Belgium

**Keywords:** working memory, executive functioning, parent–child interaction, teacher–child interaction, emotional support

## Abstract

Working memory, used to temporarily store and mentally manipulate information, is important for children’s learning. It is therefore valuable to understand which (contextual) factors promote or hinder working memory performance. Recent research shows positive associations between positive parent–child and teacher–student interactions and working memory performance and development. However, no study has yet experimentally investigated how parents and teachers affect working memory performance. Based on attachment theory, the current study investigated the role of parent and teacher emotional support in promoting working memory performance by buffering the negative effect of social stress. Questionnaires and an experimental session were completed by 170 children from grade 1 to 2 (*M*_age_ = 7 years 6 months, *SD* = 7 months). Questionnaires were used to assess children’s perceptions of the teacher–student and parent–child relationship. During an experimental session, working memory was measured with the Corsi task backward (Milner, 1971) in a pre- and post-test design. In-between the tests stress was induced in the children using the Cyberball paradigm (Williams et al., 2000). Emotional support was manipulated (between-subjects) through an audio message (either a weather report, a supportive message of a stranger, a supportive message of a parent, or a supportive message of a teacher). Results of repeated measures ANOVA showed no clear effect of the stress induction. Nevertheless, an effect of parent and teacher support was found and depended on the quality of the parent–child relationship. When children had a positive relationship with their parent, support of parents and teachers had little effect on working memory performance. When children had a negative relationship with their parent, a supportive message of that parent decreased working memory performance, while a supportive message from the teacher increased performance. In sum, the current study suggests that parents and teachers can support working memory performance by being supportive for the child. Teacher support is most effective when the child has a negative relationship with the parent. These insights can give direction to specific measures aimed at preventing and resolving working memory problems and related issues.

## Introduction

The ability to regulate and control one’s behavior, thoughts and emotions, also referred to as executive functioning (EF), is essential in making goal-directed behavior possible (Best and Miller, 2010; Zelazo and Carlson, 2012; Diamond, 2013). Three cognitive processes are considered to form the base of EF, namely working memory, inhibition and cognitive flexibility (Miyake et al., 2000; Huizinga et al., 2006; Best and Miller, 2010; Blair et al., 2011; Zelazo and Carlson, 2012; Diamond, 2013). Previous research has shown the importance of EF in variety of life domains, including education (Diamond, 2013). For example, children with well-developed EF have more positive work habits, higher engagement in learning, lower levels of inattention, positive relationships with classmates and higher academic achievement (Brock et al., 2009; Welsh et al., 2010; Best et al., 2011; Vuontela et al., 2013). Because of the importance of EF, understanding which factors influence EF performance can provide useful insights for the prevention and intervention of EF difficulties and related problems. Recent research indicates that positive interactions with both parents (Blair et al., 2011; Hughes, 2011) and teachers (Berry, 2012; Hamre et al., 2014; de Wilde et al., 2015) can promote EF quality. However, little is known about why this is the case. This study examines the role of parents and teachers as external stress regulators by means of offering emotional support to children in a stressful situation, as one particular mechanism through which positive parent–child and teacher–student interactions can promote children’s EF performance. The study focusses on a particular aspect of EF, namely working memory. This component of EF starts to develop very early and forms an important base for other EFs, such as cognitive flexibility or planning (Diamond, 2013). Additionally, of the three core EFs, working memory has been most consistently linked to children’s general development and learning (Bull and Lee, 2014; Vandenbroucke et al., 2017).

### Working Memory and Its Development

Working memory is a limited capacity, multicomponent memory system that is capable of holding and processing information over a short period of time (Baddeley, 1986). For example, working memory is used when trying to follow multi-step instructions, which requires remembering and updating information while completing the task. Working memory is essential in a large number of activities and has often been linked to learning and learning-related behavior (e.g., Gathercole et al., 2007; De Smedt et al., 2009; Alloway and Alloway, 2010; Zheng et al., 2011; Fitzpatrick and Pagani, 2012; Desoete and De Weerdt, 2013).

Working memory starts to develop in the first year of life and continues to develop at least until adolescence (Gathercole et al., 2004; Reznick et al., 2004; Conklin et al., 2007; Diamond, 2013). The development is characterized by alternating periods of rapid and more continuous growth, with a first important developmental spurt occurring between the ages of 2–8 (Hongwanishkul et al., 2005; Ganea and Harris, 2013; Kibbe and Leslie, 2013; Moher and Feigenson, 2013). This developmental pattern clearly shows parallels with the development of the prefrontal regions of the brain (Anderson, 2002). However, despite the clear importance of biological maturation processes in working memory development, the frontal brain regions and its related cognitive processes are characterized by plasticity and are sensitive to environmental stimulation, especially during periods of rapid growth (Anderson, 2002; Huttenlocher, 2002). The current study focusses on children at the beginning of primary school (ages 6–8), an age group that falls within the first period of strong development.

### Adult–Child Interactions at Home and at School as Developmental Contexts

The role of environmental factors for working memory performance and development has been far less researched compared to biological aspects (Hughes, 2011). Most studies available to date focus on the home environment and parent–child interactions. These studies show that positive factors in the home environment can promote working memory development, while negative factors can hinder the development of this core EF (see Hughes, 2011 for a short overview). The quality of the interaction between parents and their children is one such important promoting factor within the home environment. For example, the affective quality of parent–child interactions has an influence on working memory as indicated by studies showing that higher levels of parental support (Schroeder and Kelley, 2009), maternal sensitivity and autonomy support (Bernier et al., 2010) and maternal positive engagement (Rhoades et al., 2011) predict higher working memory performance. On the other hand, more negative intrusiveness by the mother predicts lower working memory performance (Rhoades et al., 2011). In sum, parents who interact with their children in a positive and supportive way can promote their children’s working memory development, while negative interactions can hinder this development.

More recently, researchers started focusing on the role of the school and classroom environment as an important developmental context for EF and working memory. Particularly, the affective quality of teacher–student interactions is an important influencing factor for working memory in children. The quality of the teacher–student relationship has mainly been viewed from an attachment perspective, which focusses on the importance of closeness, conflict and dependency in the relationship for children’s development (Verschueren and Koomen, 2012; Settanni et al., 2015). A study of Hamre et al. (2014) showed, for example, that in classes with more sensitive teachers, children performed better on a working memory task. Another study suggests that the affective quality of the dyadic teacher–student relationship, rather than classroom level interactions, is important for later performance on an EF task including a working memory component (Cadima et al., 2016). Teacher–student closeness appears to be positively related to children’s working memory (Cadima et al., 2016), while conflict has a negative association with working memory performance (de Wilde et al., 2015). Overall, the higher the levels of positive affect between a child and its teacher, the better the child’s working memory performance and the higher the levels of negative affect between a child and its teacher, the worse children’s working memory performance.

Despite the increasing evidence for the importance of parent–child and teacher–student interactions for working memory performance our understanding is still limited. First, previous studies examining how parent–child and teacher–child interactions relate to working memory are correlational in nature. As a consequence, it is unclear whether this relationship is causal or that additional variables confound this relationship. The current study attempts to contribute to this gap by experimentally manipulate emotional support and examine the effect of this manipulation on children’s working memory. Second, little is known about the mechanisms underlying this relationship. The current study therefore explores the role of one plausible mechanism, offered by the attachment-theory, namely the buffering effect of parents and teachers emotional support when the child experiences distress.

### The Buffering Role of Adult–Child Attachment Relationships in Stressful Situations

Attachment refers to the deep and enduring affectionate bond between a child and a significant adult (Bowlby, 1969). In the early years of life children form an attachment bond with their primary caregivers (Bowlby, 1969). Evidence now suggests that other significant adults, such as teachers, can also function as an attachment figure (Commodari, 2013). Verschueren and Koomen (2012) argue that the bond between a child and its teacher cannot be considered fully equal to the bond between a child and its primary caregiver as it is (in most cases) not enduring and exclusive and the teacher’s role is primarily instructional rather than focused on emotional investment. Yet, there are similarities between the parent–child and teacher–child bond, including the importance of sensitivity in predicting the quality of this bond (Ahnert et al., 2006; Verschueren and Koomen, 2012), the display of attachment-related behaviors of the child toward the adult, and the occurrence of similar classifications of attachment-related behaviors (Ahnert et al., 2012). Teachers can thus be seen as ad hoc attachment figures (Verschueren and Koomen, 2012).

When children form a positive bond with significant adults, characterized by high levels of warmth and low levels of conflict, they will display two types of attachment behaviors. Both may enhance working memory performance and development. First, as children feel confident and have trust in their caregivers, they will explore their environment independently and engage more in stimulating and challenging activities at home or in the classroom (O’Connor and McCartney, 2007; Roorda et al., 2011; Commodari, 2013). The caregiver functions as a secure base. This is likely to provide children with more frequent and more challenging opportunities to practice their working memory skills. Second, during moments of distress the child will return to the caregiver and look for comfort, which will reduce the child’s levels of stress (Verschueren and Koomen, 2012; Commodari, 2013). The caregiver functions as a safe haven. Both the quality of parent–child and teacher–student relationships have been previously linked to stress and stress regulation (Blair et al., 2011; Ahnert et al., 2012), while other studies have shown a negative impact of stress on working memory performance and development (e.g., Evans and Schamberg, 2009; Blair et al., 2011; Hanson et al., 2012). Parents and teachers can thus function as external stress regulators and as such provide children with a more appropriate environment for working memory development.

Although these attachment mechanisms are plausible and some studies partially provide support for them, no study has, to our knowledge, directly tested such mechanisms for EF. The current study therefore attempts to broaden our understanding in these underlying processes by directly examining one potential mechanism, namely parents and teachers as an external stress regulators (safe haven mechanism).

### Current Study

The aim of the current study is to enhance our understanding of the association between parent–child and teacher–student relationships, on the one hand, and working memory performance, on the other. In an experimental design, the effect of parents and teachers emotional support on children’s working memory performance is investigated, while examining the buffering of stress as a potential underlying mechanism. Specifically, after stress is induced through an experimental manipulation, children will hear a neutral message (weather report) or a supportive message of an unfamiliar person, a parent or the teacher. It is expected that stress will result in decreased working memory performance when children hear a neutral message (Hawes et al., 2012). A supportive message from parents and teachers is hypothesized to decrease the induced stress and therefore a stable working memory performance is expected in these conditions (Blair et al., 2011; Ahnert et al., 2012). Such a buffering effect is not expected when children hear a supportive message from a stranger, as the effect is expected to result from the interpersonal bond, rather than the positive nature of the message. Additionally, it can be expected that the positive effects of parent and teacher support will be more pronounced when children have a positive relationship with the parent or teacher, as children then rely more on the parent or teacher for comfort when distressed (a safe haven; Roorda et al., 2011; Verschueren and Koomen, 2012).

## Materials and Methods

### Participants

Seven regular schools for primary education, located in three provinces in Belgium, agreed to participate in the current study. In these schools, the teachers of all first and second grade classrooms were asked for their collaboration in the current study. This resulted in 18 participating classrooms (66.7%). Fifteen classrooms (83.3%) had a female teacher. Teachers handed out information letters and informed consents to the parents. Written informed content was obtained from 205 parents (56.6% participation rate). Consent was provided by the primary caregiver. If parents were divorced and had a co-parenting arrangement, both parents gave their consent for participation. Due to time constraints data could not be fully collected for all children. Therefore, the experiment was conducted in a subsample of children, which were randomly selected. In the end, 170 children participated in the experiment. There was no drop-out during the experiment: children who started the experimental session, always finished it.

The sample consisted of 43 first grade children (6 classrooms), 100 s grade children (10 classrooms) and 24 children in mixed grade classrooms (2 classrooms). Children were between 6 years 3 months and 9 years 1 month (*M* = 7 years 6 months, *SD* = 7 months) when the experiment was conducted. Background characteristics of the sample were reported by the parents (cf. 2.3.1) and an overview can be found in **Table 1**. The sample is representative for the average population in Flanders with regard to the parents’ employment status (5.1% unemployment, 73.3% employment; Eurostat 2015). However, the sample includes more highly educated primary caregivers than the population in the region of Flanders (37.2%; Eurostat 2015) and most families have a higher monthly net income compared to the average in Flanders (2689,58 euros; Statistics X 2014). The current sample mostly consisted of typically developing children (*n* = 165), though parents of 22 children reported psychosocial problems of their child. From these, six children were reported to have a disorder; three children with an Attention-Deficit/Hyperactivity Disorder (ADHD) and three children with an Autism Spectrum Disorder (ASS). None of the parents reported physical health problems or medication use that could influence data collection.

**Table 1:** Distribution of background characteristics of the participants who completed the experiment (*n* = 170).

Characteristics	Sample	
	*n*	%
Boys	89	43.3
Primary caregivers with at least a Bachelor’s Degree	98	65.8
Work status primary caregiver		
Working ≥ 75%	99	66.4
Working < 75%	21	14.1
Not working, voluntary	21	14.1
Not working, involuntary	8	5.4
Monthly net family income		
<1000 euros	1	0.7
1000–2000 euros	18	12.2
2000–3000 euros	23	15.5
3000–4000 euros	38	25.7
4000–5000 euros	42	28.4
>5000 euros	26	17.6
Mother tongue		
Monolingual Dutch speaking	133	86.9
Bilingual Dutch speaking	6	3.9
Other languages	14	8.4
Parents with Belgian nationality		
Both parents	136	84.5
One parent	13	8.1
No parent	12	7.5
Child with Belgian nationality	143	93.5

### Instruments

#### Demographics

Parents filled out a self-constructed questionnaire to report on a number of background characteristics of the participating child and their family. First, parents provided socioeconomic information by indicating the caregivers’ educational level, occupational status and monthly net income. The educational level was recoded into low-educated (i.e., a degree of secondary education at most) and highly educated (i.e., at least a Bachelor’s Degree). Occupational status was recoded into full-time working (i.e., working at least 75%), part time working (i.e., working less than 75%), voluntarily not working (i.e., housewife or houseman, on pension, maternity leave and temporary career breaks for more than 3 months) and involuntarily not working (i.e., in search of employment or unfit for work). Family monthly net income was categorized as below 1000 euros, between 1000 and 2000 euros, between 2000 and 3000 euros, between 3000 and 4000 euros, between 4000 and 5000 euros and above 5000 euros. Second, parents gave information about the physical and psychosocial health and medication use of the participating children. Finally, the nationality and mother tongue of the participating child and the caregivers was reported.

#### Teacher–Child and Parent–Child Relationship

To assess children’s perception of the quality of their relationship with the teacher, the Young Children’s Appraisals of Teacher Support (Y-CATS; Mantzicopoulos and Neuherth-Pritchett, 2003; Spilt et al., 2010) was used. This scale consists of 27 statements about the relationship between the child and the teacher. The researcher reads each statement and the child places the card with the statement in a safe when it is true and in a trashcan when it is untrue. This approach is first practiced with two example items: one that is clearly true (‘my teacher is bigger than me’) and one that is clearly untrue (‘my teacher has blue hair’). The Y-CATS has three subscales, namely warmth (11 items, e.g., ‘My teacher says nice things about my work’), conflict (10 items, e.g., ‘My teacher gets angry with me’) and autonomy support (6 items, e.g., ‘My teacher lets me do things I like’). Scores are calculated for each scale by summing the scores of the respective items. The Dutch version of the Y-CATS has an acceptable to satisfactory internal consistency in previous studies, with Cronbach’s alphas of 0.65, 0.72, and 0.61 for warmth, conflict and autonomy support, respectively (Spilt et al., 2010). In the current study, items 23 and 27 (Warmth Subscale), 22 (Conflict subscale) and 3 (Autonomy Support subscale) were deleted because of negative or extremely low item-rest correlations. The final Cronbach’s alphas in the current study of the subscales were 0.90, 0.79, and 0.52 respectively. The internal consistency of Autonomy Support was unsatisfactory in the current sample and could not be further raised by deleting specific items. This subscale was therefore not used in further analyses. Additionally, a dichotomous score was calculated categorizing each participant as low or high on each subscale. Children were categorized as high with a score higher than four for both warmth and conflict. This means that for at least half of the items, presence was indicated by the child (i.e., the item was put in the safe).

Children’s perception of their relationship with their primary caregiver was assessed with the Parent–Child Interaction Questionnaire-Revised child version (PACHIQ-R; Lange et al., 2002). The original scale consists of 25 statements which children have to evaluate on a 5-point scale. However, because of the young age of the children in the current sample, the same administration procedure was used as with the Y-CATS, reducing the response possibilities to a true or false choice. Children completed the questionnaire for the parent who indicated to be the primary caregiver (83% mothers). The items of the PACHIQ-R child version were originally found to be best described in two subscales, an Acceptance scale (8 items, e.g., ‘If I’m sad about something, my mother comforts me’) and a Conflict resolution scale (17 items, e.g., ‘Most of the times, I do what my mother asks’). However, given the changes in procedure and the younger age sample the structure of the questionnaire was reexamined in the current sample. To this end, Exploratory Factor Analysis was conducted, using Parallel Analysis (Horn, 1965) to determine the number of factors to extract. This method compares the observed eigenvalues of the factors with the eigenvalues of a series of simulated data matrices with the same characteristics. This method is more conservative than the ‘eigenvalue-greater-than-one’ criterion and results less often in an overestimation of the number of factors to be extracted. The default number of 100 simulations and 95th percentile of the eigenvalues were used. Results indicated a three-factor structure was more appropriate for the current sample. The first subscale was Warmth in the parent-child relationship (9 items; e.g., ‘When I do something for my mother, I can tell that she likes it’). The second subscale was Conflict (9 items, e.g., ‘Whatever my mother tells me, I do what I want’). Sensitivity was the final subscale (6 items; e.g., ‘When I am sad, my mother comforts me’). A score was calculated for each subscale by summing the items of the respective scale. Item 19, belonging to the Sensitivity subscale, was deleted due to a low correlation with the rest of the scale. Cronbach’s alphas in the current study were acceptable to good (0.81, 0.69, and 0.60). Scores were calculated for each subscale by summing the score on each item. Again, a low-high dichotomization was made. Children who scored higher than four on warmth, higher than four on conflict and higher than three on sensitivity were categorized as high on the respective subscale. As only eight children were categorized in the low sensitivity group, parent-sensitivity was excluded from further analysis.

#### Working Memory

To assess working memory a backward version of the Corsi blocks test (Milner, 1971) was used. Children were presented with a wooden board with nine irregularly spaced blocks. The experimenter tapped a series of blocks, at a rate of one block per second, and the child was asked to repeat the sequence in the reverse order. A standardized procedure was used. After verbal instructions given by the researcher and two practice items, children started the test with the reproduction of a sequence of two blocks. After four correct items, difficulty was increased with one block, until a maximum of nine blocks per sequence was reached. When a child was unable to reproduce three sequences of the same difficulty the test ended and the researcher continued with the rest of the experiment. Two parallel sets of items were used, one with items from the WMTB-C (Gathercole and Pickering, 2000) and one with items from the Automated Working Memory Assessment (AWMA; Alloway, 2007). The difficulty of items (based on the number of crossings that in the pathway of the sequence; Busch et al., 2005) was evaluated in advance and both set of items were comparable in difficulty. Order of the two sets of items was counterbalanced; half of the children received the WMBT-C items as pre-test and half of the children received AWMA-items as pre-test. A span score was recorded as the highest number of blocks that could be reproduced by the child in reverse order. An item score was calculated as the number of sequences correctly reproduced by the child. Both scores were highly correlated (*r* = 0.92), therefore, in further analysis, the item score was used as a measure of working memory performance. This type of score is often used for tasks measuring working memory performance (Gathercole and Pickering, 2000; Alloway, 2007).

#### Stress Induction

To induce stress, the Cyberball paradigm was used (Williams et al., 2000). This paradigm simulates online social exclusion and causes mild general distress and increased physiological arousal (Abrams et al., 2011; Kelly et al., 2012). Children are told they will play a ball throwing game online with two other children. In reality the two other players are not real. The game is programmed in such a way that the participant is included during the first 18 throws, when each player receives the ball one third of the throws. However, he or she is excluded by the two fictive players during the last 20 throws. All players are represented by avatars and fictive names are mentioned for the two opponents with whom the participant is playing the game (one boy’s name and one girl’s name). For ethical reasons, all children play an inclusion version at the end of the experiment, with 18 trials and each player receiving the ball one third of the time. Although Cyberball is known as a mild stressor, previous research showed that this manipulation of social exclusion induces sufficient distress to negatively impact working memory performance in children (Hawes et al., 2012). After the game, children indicated how often they received the ball from the other players (never, sometimes, often, or always) as a manipulation check.

#### Emotional Support

Emotional support offered by the parent or teacher was manipulated by means of an audio recording. An audio message has previously been used in attachment research and has an effect on children’s oxytocin levels, which are related to the display of attachment related behaviors (Seltzer et al., 2010). Children either heard a weather report, a supportive message from an unknown person, a supportive message from their parent or a supportive message from their teacher. The content of the three supportive messages was standardized (Appendix A). All messages lasted approximately 30 s. The message provided by the parent was always a message from the primary caregiver as indicated by the parent(s) (75% mothers). Teachers that provided the message were primary teachers (86% female) who taught all courses to the children, whit the exception of physical education and religion. A blocked randomization was used for assigning children to the four conditions, to ensure that conditions were equally divided over schools, classrooms and gender (Suresh, 2011). At the end of the experiment the child indicated how much he or she liked receiving the audio message (not at all, not really, doesn’t matter, somewhat or very much).

### Procedure

This study was approved by the Social and Societal Ethics Committee of the University of Leuven. In the first part of the study children completed two questionnaires to assess their perception on the relationship with their parent and teacher. The assessment was completed during an individual session of approximately 20 min in a quiet room at school. The researcher read the statements of the questionnaires out loud and the child indicated whether they were true or false. On the same day demographic questionnaires were given to the parents. Parents returned the completed questionnaire 1 week later. In the second part of the study, the experiment was conducted during an individual session with the child. On average the experimental session was completed 26 days after the administration of the child questionnaires. The experimental session lasted approximately 30 min and was conducted in a quiet room at school. During this session, children first completed a working memory task (pre-test). This was followed by a stress induction through a computer game and a manipulation check. After the game, children heard one of four audio messages: a weather report, a supportive message of an unknown, a supportive message of a parent or a supportive message of the teacher. This audio message was used to manipulate the emotional support offered by the parent or teacher. The stranger condition was added in order to distinguish whether the effect on working memory was due to the positive tone of the message or the positive interpersonal relationship with the person giving the support. A parallel version of the working memory task was then used to assess post-test working memory performance. For ethical reasons, the session finished with a non-stressful version of the computer game and children were debriefed about the true meaning of the game. None of the children refused to play the final game. Children received an age-appropriate reward for their participation in the study.

### Analyses

Descriptive statistics were calculated for the working memory outcomes and the manipulation checks for both the stress induction and the audio message. Additionally, *t*-tests, ANOVAs and correlational analyses were conducted to examine whether gender, Corsi test version, socioeconomic background (parents educational level, working status and family income) and age were significantly related to pre-test working memory scores. Finally, before conducting the main analyses, it was examined whether pre-test working memory significantly varied between classrooms, which would indicate multilevel analysis would be needed to control for children being nested within classrooms. A two-level null random intercepts model was calculated in MLWin 2.1 (Rasbash et al., 2009), showing that there was only significant between-subject variance (σ = 0.67, *SE* = 0.08, χ^2^ = 74.95, *p* < 0.001) and no significant between classroom variance (σ = 0.08, *SE* = 0.05, χ^2^ = 2.382, *p* = 0.123). Traditional analysis were thus preferred above multilevel analysis. These preliminary analyses were followed by the main analyses. Repeated measures ANOVAs were conducted to examine the effect of parent and teacher support after stress induction on changes in working memory performance. Pre- and post-test scores of the Corsi task were used as within-subject variable and condition as between-subject factor. Analyses were controlled for relevant background characteristics of the participants. Finally, additional repeated measures ANOVAs were conducted adding the quality of the parent–child and teacher–student relationship as dichotomous between-subject factors. This allowed us to examine whether the effect of the conditions depended on this relationship quality. All analyses are conducted in SPSS (IBM Corp., 2013).

## Results

### Descriptives

**Table 2** shows the means and standard deviations of the scales measuring the parent–child and teacher–student relationship and of the working memory outcomes, as well as the correlations between these variables. Parent–child and teacher–student warmth were highly correlated, while a medium correlation existed between parent–child and teacher–student conflict. **Table 3** shows the descriptive statistics for the working memory outcomes in the different conditions. There are no significant differences between the conditions in pre-test scores.

**Table 2:** Means and standard deviations of and correlations between the parent–child and teacher–child relationship scales (non-dichotomized), and working memory outcomes (*n* = 170).

	1	2	3	4	5	*M* (*SD*)
(1) Parent warmth						6.97 (2.31)
(2) Parent conflict	-0.46***					1.99 (1.98)
(3) Teacher warmth	0.66***	-0.37***				6.96 (2.76)
(4) Teacher conflict	-0.69***	0.47***	-0.68***			3.20 (2.50)
(5) pre item score	-0.16*	-0.18*	-0.07	-0.03		16.29 (4.86)
(6) post item score	-0.22**	-0.21**	-0.18*	0.08	0.67***	16.44 (4.56)

*^∗^*p* < 0.05, ^∗∗^*p* < 0.01, ^∗∗∗^*p* < 0.001.*

**Table 3:** Descriptive statistics of the working memory outcomes within and across conditions.

	Item score	
	Pre	Post
**Condition**	***M* (*SD*)**	***M* (*SD*)**

Weather report	16.75 (5.14)	16.11 (4.35)
Stranger support	15.72 (4.62)	15.77 (4.85)
Parent support	16.67 (5.15)	17.35 (4.77)
Teacher support	16.02 (4.62)	16.58 (4.27)
Total	16.29 (4.86)	16.44 (4.56)

As a manipulation check, after the Cyberball game children were asked how often they had received the ball (never, sometimes, often, or always). Most children indicated they received the ball sometimes (86.5%), often (8.2%) or never (3.5%) and thus experienced exclusion to some extent. However, three children (1.8%) indicated they always received the ball. These three children were removed for further analyses.

Additionally, a manipulation check was conducted to examine to what extend the children liked the audio message they received. As expected, the supportive message of the parent, teacher or stranger was liked very much (52.5, 48.8, and 35.7% respectively) or somewhat liked (40.0, 34.9, and 45.2%) by most children. The weather report was somewhat liked (31.8%) or did not really matter (43.2%) for most children. This indicates that the supportive message was successful and positively received by the children.

### Preliminary Analyses

Children’s working memory performance was not related to gender. Additionally, both versions of the Corsi task could be considered parallel versions, as indicated by the lack of a significant difference in working memory score at pre-test. Finally, age was significantly correlated with the pre-test working memory score (*r* = 0.38; *p* < 0.001). Child gender and order of the Corsi tests was therefore not taken into account, whereas all analyses controlled for age effects.

With regard to children’s socioeconomic background, the educational level of the primary caregiver was related to working memory at pre-test [*t*(147) = -4.10; *p* < 0.001], with children of highly educated parents performing better. Similarly, a positive relationship was found between families’ monthly net income and pre-test working memory performance (Spearman ρ = 0.23, *p* = 0.005). Finally, the work status of the primary caregiver was related to the working memory score [*F*(3,145) = 3.21; *p* = 0.025]. Children of which the primary caregiver worked full-time (*M* = 16.95) or stayed at home voluntarily (*M* = 16.24) outperformed children of parents who were unemployed or unfit for work (*M* = 12.00). These characteristics were added as control variables in further analyses. Educational level and work status of the second caregiver were not related to working memory.

### The Effect of Emotional Support

Using repeated measures ANOVA, the changes in working memory performance from pre- to post-test in the different conditions were tested, while controlling for age, primary caregiver education level, work status and family income. No significant time × condition interaction was found, [*F*(3,135) = 0.85, *p* = 0.471] indicates that the change in working memory from pre- to post-test did not differ between the conditions.

### Moderating Effect of Parent–Child and Teacher–Student Relationship Quality

Additional repeated measures ANOVAs were performed in order to examine whether the effect of emotional support on working memory was moderated by the parent–child and teacher–student relationship quality. To this end, the dichotomized warmth and conflict scales were entered as between-subject variables.

Results show changes when adding the quality of the parent–child and teacher–student relationships. First of all, the change in working memory from pre- to post-test became significant [*F*(1,110) = 5.80, *p* = 0.018, η^2^ = 0.050], showing a small drop in working memory performance across conditions, after stress was induced.

**FIGURE 1:**
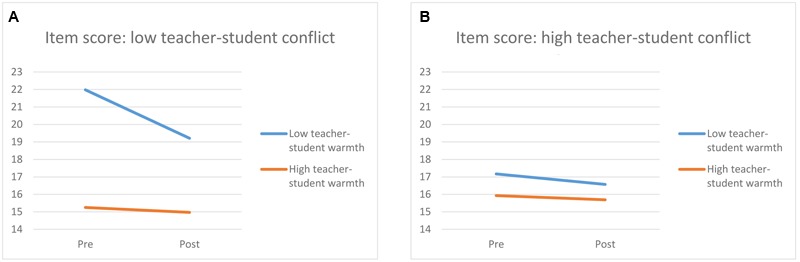
**Changes in pre-posttest working memory score for children experiencing high and low levels of teacher-student warmth in combination with low levels of teacher–student conflict (A)** or high levels of teacher–student conflict **(B)**.

Additionally, several relationship variables interacted with working memory performance. First, a time × parent–child conflict interaction [F(1,110) = 6.99, *p* = 0.009, η^2^ = 0.060] showed that children who experienced high parent–child conflict showed a decrease in working memory performance after stress induction, while children experiencing low levels of parent–child conflict did not. Second, a significant time × teacher warmth × teacher conflict interaction was found [*F*(1,110) = 5.21, *p* = 0.024, η^2^ = 0.045], shown in **Figure 1**. For children experiencing low levels of teacher–student conflict (**Figure 1A**), a decrease in working memory could be seen when there were low levels of teacher-student warmth, while working memory was stable when there were high levels of teacher–student warmth. When children experienced high levels of conflict (**Figure 1B**), working memory performance was stable irrespective of the levels of teacher–student warmth.

**FIGURE 2:**
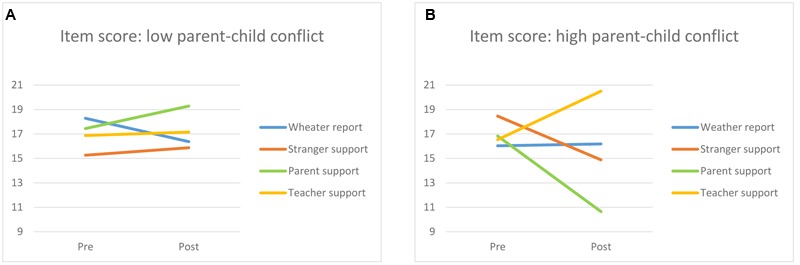
**Changes in pre-posttest working memory score for each condition (weather report, stranger support, parent support and teacher support) for children experiencing low levels (A)** and high levels **(B)** of parent–child conflict.

**FIGURE 3:**
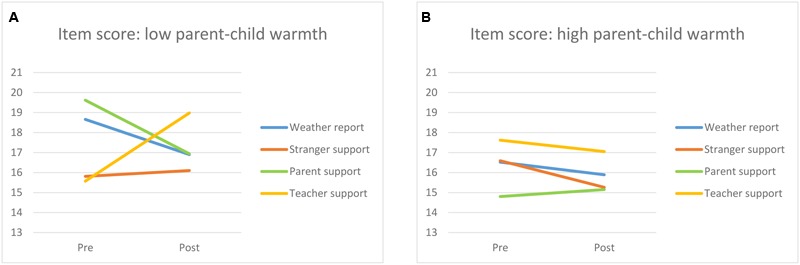
**Changes in pre-posttest working memory score for each condition (weather report, stranger support, parent support, and teacher support) for children experiencing low levels (A)** and high levels **(B)** of parent–child warmth.

Finally, two interactions were found indicating that the effect of the conditions on working memory performance depended on the quality of the child–parent interaction. First, there was a medium sized time × condition × parent conflict interaction [*F*(3,110) = 2.99, *p* = 0.034, η^2^ = 0.075]. As can be seen in **Figure 2**, the audio message made almost no differences when children experienced low levels of conflict with the parent (**Figure 2A**). However, when children experienced high levels of conflict with the parent their performance decreased after hearing a supportive message from a parent or from a stranger, whereas it increased when hearing a supportive message from the teacher (**Figure 2B**). *Post hoc* analysis indicate that for children experiencing high levels of parent–child conflict, there were no differences in working memory performance at pre-test, while at post-test the difference between children supported by teachers and children supported by parents was just above significance [*t* = -8.50, 95% CI = [-17.08; 0.08], *p* = 0.052]. For children experiencing low levels of parent-child conflict, *post hoc* analysis revealed no differences at both pre- and post-test. Finally, a similar result was found for parent–child warmth, with a three way time × condition × parent–child warmth interaction [*F*(3,110) = 3.78, *p* = 0.013, η^2^ = 0.093]. Children experiencing high levels of warmth seemed not to be affected by the different audio messages (**Figure 3B**). Children experiencing low levels of warmth from the parent experienced a negative effect of parental support, while teacher support resulted in increased working memory performance (**Figure 3A**). Post hoc analysis indicated that for children experiencing high levels of parent–child warmth, there were no differences between conditions at pre- and post-test. For children experiencing low levels of parent-child warmth, children in the teacher support condition scored significantly lower at pre-test compared to the children in the parent support condition (*t* = 5.24, 95% CI = [0.75; 9.74], *p* = .024) and these differences were no longer visible at post-test (*t* = 1.15; 95% CI = [-3.09; 5.39], *p* = 0.585).

## Discussion

Previous research has shown that a positive parent–child or teacher–student affective relationship can support EFs and working memory. Whereas these previous studies were all correlational in nature, the current study attempted to experimentally demonstrate the effect of parent and teacher support on working memory performance. Additionally, this study examined whether the effect of parent and teacher emotional support can be seen as a stress-buffering effect. This is, to our knowledge, the first study that tries to uncover the reason why parents and teachers can promote working memory performance through a positive relationship.

### The Effects of Stress on Working Memory Performance

It was expected that after a stress inducing game, children’s working memory performance would decrease if they heard a neutral message afterward. In contrast to what we had expected (based on Hawes et al., 2012), there was no general negative effect of stress on working memory performance as shown by a drop in working memory in the weather report condition. As a consequence, the effects of emotional support of parents and teachers that were observed, cannot be linked to the underlying stress mechanism, which this study was trying to test.

A decrease after stress induction was observed in specific subgroups of children, namely children who experienced low levels of parent–child conflict, low levels of parent–child warmth, and low levels of teacher–student warmth especially in combination with low levels of teacher–student conflict. Children may be differentially susceptible to stressors and this can be influenced by different factors, such as genetics (Ising and Holsboer, 2006), gender (Hawes et al., 2012) or the quality of the parent–child and teacher–student relationship (Bernier et al., 2010; Ahnert et al., 2012).

It should be noted that the neutral message may have distracted children and reduced children’s stress levels even though it was used as a control condition. Alternatively, if children did not experienced the exclusion from Cyberball, they may have had an increase in working memory due to a learning effect. This means that a stable working memory performance after the Cyberball game might indicate a negative effect of stress if it was compared to a no stress condition. In both cases the true impact of stress and working memory might be underestimated in the current design. The addition of an objective stress measure (e.g., skin conductance or a salivary cortisol measure) or a no-stress condition may help to assess the true effect of stress on working memory performance.

### Effect of Parent and Teacher Support on Working Memory Performance

When children have a positive relationship with their parent, no clear effect of parent support was found. Results do suggest that when children have a more negative relationship with their parent (low warmth, high conflict), support offered by the parent has a negative effect on working memory performance. On the other hand, support offered by teachers has a positive effect on working memory performance when children have a negative relationship with their parent. As a result children who had a negative relationship with their parent and who heard a supportive message from the teacher outperformed or caught up with children who heard a supportive message from the parent at post-test. This indicates that teacher support might compensate for the adverse effects of a negative parent–child relationship. Such a compensating effect has previously been shown for children’s behavior with high levels of teacher warmth related to decreases in children’s aggressive behavior only for children who were insecurely attached to their mother (Buyse et al., 2011). In their review McGrath and Van Bergen (2015) indicate different explanations for the fact that a positive teacher-student relationship may compensate for other risk-factors such as a negative parent-child relationship. One possibility is that when children receive adequate support from the teacher, they will form a less negative internal working model and thus have less negative beliefs about the world and the self (Buyse et al., 2011; McGrath and Van Bergen, 2015).

The lack of effect of emotional support for children who do have positive parent–child relationships may indicate that when children are used to positive stimulation from the teacher, they need a stronger reinforcement than a short audio message to see an effect on working memory performance. Another possibility is that children with negative parent–child relationships rely more on the teacher for helping to regulate their stress levels and emotions and that these children are more easily affected by positive support from their teacher (McGrath and Van Bergen, 2015). This result is in line with broader research indicating limited effects of teacher–child relationships on children’s behavior when there is already a positive parent–child relationship (e.g., Buyse et al., 2011). With this respect our results support the academic-risk hypothesis (Hamre and Pianta, 2001) stating that the quality of teacher–child relationships are most important for those children at risk for negative school adjustment, because they have more to gain or to lose than other students (Roorda et al., 2011).

Finally, the negative effect of parent support for children with negative parent–child relationships was an unexpected finding that warrants some attention. This might be explained by the fact that children build internal working models of attachment, mental schemes containing information about social relationships, based on experiences with early attachment figures (Dykas and Cassidy, 2011). Children use these internal working models to store information about previous social experiences and to form expectations about how future social experiences will be like. When children do not have a positive relationship with their parent they are likely to form an insecure attachment script or a negative internal working model. As a result, they are more likely to interpret social information, such as an audio message from the parent, in a negative way or they completely ignore it (Dykas and Cassidy, 2011). Also, children who have a negative bond with their parent in general respond to distressing situations with maladaptive coping strategies, which can further enhance negative feelings that are already present (Grossmann and Grossmann, 1991). Hearing a supportive message from the parent may thus have further increased children’s stress levels.

An important note should be made with regard of the impact of the observed effects. Children who experience high parent–child conflict can processes one additional item in working memory after hearing a supportive message from their teacher. In developmental research examining growth in working memory, such an increase corresponds to approximately 2 years of development (Alloway, 2011). Although effect sizes indicate small to medium effects, it should thus be taken into account that in practice the impact of the environment is substantial and might have considerable implications for children’s learning.

### Strengths and Limitations

The current study contributes to the literature in several ways. First, whereas previous studies had established relationships between parent–child and teacher–student relationship quality and working memory performance, none of the previous studies has done so in an experimental design. The current study is therefore the first that can show a causal effect of parent and teacher emotional support on working memory performance. Second, research examining the parent and teacher influences on EF has evolved independently and it was therefore previously unclear what the relative contribution of both is. The current study showed that parent and teacher influences interact with each other.

Some limitations of the current study warrant attention when interpreting the findings of the study. First, the main limitation of the current study is that, due to the lack of a no-stress condition or an objective stress measure, the effect of stress on working memory is hard to interpret. As a consequence we cannot link the effect of emotional support from parents or teachers directly to children’s stress levels. Based on previous research it is assumed that the Cyberball manipulation provides mild distress (Abrams et al., 2011; Kelly et al., 2012), though this did not clearly come forward in the current design. During the experiment large differences were observed in children’s response to the stress induction. Objective stress measures (e.g., skin conductance or salivary cortisol) and a no-stress condition would be helpful in directly linking the parent and teacher support to the proposed stress mechanism. However, irrespective of the lack of a clear stress effect, there are clear effects of emotional support on working memory performance, which is on its own a new and important insight when examine the role of parents and teachers in children’s EF performance and development. Second, it should be noted that although a limited number of statistical models were run in the current study, this did result in multiple individual tests. The results should thus be interpreted with caution and *p*-values should always be interpreted in combination with effect sizes. Third, the current study examined the acute effect of stress induction and parent and teacher support for working memory performance. Questions remain about whether parent and teacher support have effects in the long run through the buffering of the negative effects of stress on working memory. Finally, it should be noted that although the current study points out the importance of parents and teachers as safe havens, this does not exclude other potential mechanisms through which parents and teacher can influence working memory performance and development. Future research should therefore also consider the role of, for example, children’s increased exploration of the environment (parent and teacher as secure base; O’Connor and McCartney, 2007) and modeling (Rimm-Kaufman et al., 2009), direct stimulation (McNamara and Scott, 2001; Morrison and Chein, 2011) or scaffolding (Bibok et al., 2009; Hughes, 2011) by both parents and teachers.

## Conclusion

The current study shows that parents and teachers can have a substantial influence on children’s working memory performance by offering adequate emotional support. Although further research is needed to examine the underlying mechanisms of these effects, this thus confirm the idea that cognitive processes, such as working memory, do not merely depend on maturation, but can also be supported or hindered by environmental factors. Both clinicians (e.g., those providing working memory trainings) and teachers should thus not only pay attention to the cognitive stimulation of children, but should recognize the importance of affective factors, such as the affective quality of relationships with significant others. Being attentive to the emotional environment in which children grow up might be an important element that can complement current attempts in the prevention and intervention of working memory problems.

## Author Contributions

LV was responsible for the preparation of the study, data-collection and the writing of the current manuscript. JS designed the study, supported the data-collection and gave feedback on the analysis, the interpretation of the data and the current manuscript at multiple occasions. KV supported the data-collection and gave feedback on the analysis, the interpretation of the data and the current manuscript at multiple occasions. DB supported the data-collection and gave feedback on the analysis, the interpretation of the data and the current manuscript at multiple occasions

## Conflict of Interest Statement

The authors declare that the research was conducted in the absence of any commercial or financial relationships that could be construed as a potential conflict of interest.
