# Supplementary material for: Keeping the Spirits Up: The Effect of Teachers’ and Parents’ Emotional Support on Children’s Working Memory Performance
Source: Front Psychol. 2017 Apr 4;8:512. doi: 10.3389/fpsyg.2017.00512 (PMC5378781; doi:10.3389/fpsyg.2017.00512)
Supplement: Supplementary file 1 [file Data_Sheet_1.docx]

Appendix A. Content of the supportive audio message.

“Hi! I heard you are participating in a study and have to do all these tasks. Doing this with someone you don’t know must be exciting. Maybe the tasks are not that easy and you are a little nervous. But you don’t need to be. I am absolutely sure that you are doing a good job. Before you know it, you will be finished and you can go back to the classroom. So just continue for a little while and keep trying your best. Than it will all work out just fine! Bye!”

“Hallo! Ik heb gehoord dat je meedoet aan een onderzoek en allemaal taakjes moet maken. Zo bij iemand die je niet kent, dat zal wel spannend zijn. En als de taakjes niet zo gemakkelijk zijn, dan ben je misschien wat zenuwachtig. Maar dat is helemaal niet nodig. Ik ben er zeker van dat je het kan en dat je goed bezig bent! Voor je het weet ben je al klaar en kan je terug naar de klas. Dus doe nog even verder en blijf goed je best doen. Dan lukt het wel! Dag!”
